# The prediction and verification of outcome of extracardiac conduits fontan based on computational fluid dynamics simulation

**DOI:** 10.3389/fphys.2022.1078140

**Published:** 2022-11-24

**Authors:** Yong Zhang, Minhua Fang, Zengwei Wang, Yu Liu, Chunzhen Zhang, Zhenlong Wang, Huishan Wang

**Affiliations:** Department of Cardiovascular Surgery, General Hospital of Northern Theater Command, Shenyang, Liaoning, China

**Keywords:** functional single ventricle, total cavopulmonary connection, numerical simulation, fluid dynamics, computed tomography angiography

## Abstract

**Objective:** This study applied preoperative computed tomography angiography (CTA) and computational fluid dynamics (CFD) simulation to predicte and verify the outcome of Y-shaped extracardiac conduits Fontan for functional single ventricle.

**Methods:** Based on the preoperative CTA data of functional single ventricle (FSV), 4 types of spatial structures of extracardiac conduits were designed for 4 experimental groups: Group A, a traditional TCPC group (20 mm); Group B, a diameter-preserving Y-shaped TCPC (YCPC) group (branch 10 mm); Group C, YCPC group (branch 12 mm); and Group D, an area-preserving YCPC group (branch14 mm). Four indicators including flow velocity, pressure gradient (PG), energy efficiency and inferior vena cava (IVC) blood flow distribution were compared. The optimal procedure was applied. The radionuclide lung perfusion, CTA, echocardiography, cardiovascular angiography and catheterization were performed postoperatively.

**Results:** There were the lowest PG, the lowest flow velocity of branches, the highest energy efficiency, and a relatively balanced and stable distribution of IVC flow for group D. Subsequently, the group D, a handcrafted Y-shaped conduit (14 mm) was used for the YCPC procedure. There was no postoperative PG between the conduit and pulmonary artery with normal pressure and resistance. IVC flow was distributed uniformly.

**Conclusion:** CTA-based CFD provided more guidance for the clinical application of TCPC. A comprehensive surgical design could bring good postoperative outcome. Area-preserving YCPC has more advantages than TCPC and the diameter-preserving YCPC. The study effectively improved the feasibility of clinical applications of YCPC.

## 1 Introduction

Functional single ventricle (FSV) is a type of complex congenital malformations with only one fully functional ventricle. Due to the unique anatomical structure of ventricles, it is not clinically possible to fully reconstruct the two-ventricle series circulation through surgical operations. Only a single-ventricle series circulation can be realized by using the Fontan procedure, which completely separates the parallel systemic and pulmonary circulations to improve the clinical symptoms of hypoxia and eliminates single ventricular volume overload (Piran et al., 2002). However, the Fontan circulation is limited by its single ventricular physiology and can only rely on increasing central venous pressure to maintain pulmonary circulation, which in turn causes increased vascular resistance of multiple organs and decreased blood perfusion. In addition, the uneven distribution of blood flow of the inferior vena cava (IVC) in bilateral lungs leads to a significant increase in the incidence of pulmonary arteriovenous fistula in patients undergoing Fontan procedure (de Zelicourt et al., 2011). Long-term Fontan circulation after surgery often leads to multiple complications such as recurrent pleural effusion, protein-losing enteropathy, liver cirrhosis, and cardiac insufficiency (Dabal et al., 2014). Therefore, the efficiency of single-ventricle circulation and the uniform distribution of IVC flow after Fontan procedure directly affect the life span and quality of life of patients with FSV. Furthermore, studies have shown that different spatial structures connecting the vena cava to the pulmonary artery affect a variety of hydrodynamic parameters and directly affect the efficiency of the Fontan circulation and the uniform distribution of blood flow of the IVC (Soerensen et al., 2007; Kanter et al., 2012; Trusty et al., 2016).

Therefore, how to reduce hemodynamic energy consumption after a Fontan procedure is a problem that needs to be solved urgently in Fontan procedures. Since de Leval (Marsden et al., 2009) proposed total cavopulmonary connection (TCPC) that can produce more linear blood flow in blood vessels and lower the energy consumption in 1988, more and more research centers have carried out relevant studies (de Leval et al., 1988; Sundareswaran et al., 2012; Tang et al., 2013).

Later, a theoretically more efficient procedure that Y-shaped conduit total cavopulmonary connection (YCPC) was proposed. However, Trusty et al. Trusty et al. (2016) found that there were higher resistance and less balanced hepatic flow for commercially available YCPC than TCPC, while Martin et al. Martin et al. (2015) also found handcrafted YCPC might bring unbalanced hepatic flow.

There was still a big gap between the theoretical efficacy of TCPC and its postoperative efficacy. It is our major objective to minimize this gap. We use the computed tomography angiography (CTA) imaging data and computational fluid dynamics (CFD) simulation for surgical patients to obtain preliminary experimental data.

Artificial intelligence (AI) has been applicated in medical image analysis and deep learning for a long time. We try to quantify and score the simulation indicators to select the optimal procedure, which is convenient for machine learning. Based on this experimental basis, we hope to obtain an objective and standardized surgical design with the help of AI in the future. Therefore, this study was designed to predict and verify the outcome of Y-shaped extracardiac conduits Fontan for FSV based on preoperative CTA and CFD.

## 2 Methods

This study was approved by our institutional ethics committee (IRP approval number: 2020015, 16 April 2020) and patients’ guardians have given their consent statement for the publication of this study.

### 2.1 Image acquisition and geometric model construction

A 17-year-old boy was diagnosed preoperatively with SLL-type congenitally corrected transposition of the great arteries with right ventricular dysplasia and ventricular septal defect. Preoperative cardiovascular computed tomography angiography (CTA) was performed. Subsequently, a TCPC with extracardiac conduits was planned for the patient, and the patient’s personalized CTA data (standard Dicom format) was imported into a medical image processing software (Mimics Research, 20.0). First, the images were filtered and denoised to complete image optimization. Then, the nonrelevant images of the heart, spine and aorta were removed. The data for SVC and IVC, pulmonary artery and their branches were preserved to reconstruct three-dimensional images. The branches of pulmonary artery were extended to 50 mm to provide stability.

### 2.2 CTA-based surgical design

According to the experimental design, 4 types TCPC were designed to connect the extracardiac conduit and the pulmonary artery:

Group A (20 mmT), a conventional TCPC group: The IVC was anastomosed with the pulmonary artery *via* an extracardiac conduit with a diameter of 20 mm using the conventional method, and the anastomosis was close to the left hilum. The superior vena cava (SVC) was anastomosed with the pulmonary artery, and the anastomosis was close to the right hilum. This design of anastomosis can reduce the competition between blood flows in the SVC and IVC and thus reduce energy consumption.

Group B (20 mmY), a commercially available YCPC group (diameter-preserving 20 mm-10 mm-10 mm): The IVC was connected to the pulmonary artery *via* a Y-graft (extracardiac conduit), and the sum of the diameters (10 mm) of the two branches of the Y-graft was equal to the diameter of the trunk (20 mm); the two branches were anastomosed to the pulmonary artery close to the left hilum and right hilum, respectively, and the SVC was anastomosed to the pulmonary artery between the two branches to avoid energy consumption caused by competition between blood flows.

Group C (12 mmY), an intermediate YCPC group (20 mm-12 mm-12 mm): The trunk diameter of the extracardiac conduit remains unchanged, and the branch diameter (12 mm) was larger than one half the trunk diameter. The anastomosis method was same as that of group B.

Group D (14 mmY), a handcrafted YCPC group (area-preserving 20 mm-14 mm-14 mm): The square of the trunk radius (10 mm) of the extracardiac conduit is approximately equal to the sum of the squares of the radii (7 mm) of two branches, and the anastomosis method was same as that of group B.

### 2.3 Numerical simulation method and boundary condition settings

Based on the completion of the construction of the three-dimensional geometric model, the geometric models of the four preoperative plans were meshed. Meshing software Hypermesh was used to mesh the computational domains. Hexahedral and tetrahedral meshes were used to test the mesh sensitivity, and the adaptive mesh technology was used for local mesh refinement. (It is recommended to add the results of sensitivity analysis to confirm whether tetrahedral or hexahedral mesh should be used, the number of meshes, and the graph of the computational domain mesh changes before and after mesh adaptation.)

Using the CFD analysis software Fluent and the in-house developed three-component coupling program, the team used the Lagrangian-Euler method to carry out numerical simulation of multiple working conditions and multiple schemes.

Due to its low flow velocity and laminar flow, blood was regarded as an incompressible Newtonian fluid, and the influence of gravity on it was neglected. The walls of artificial blood vessels and pulmonary arteries were assumed to be rigid walls, and no-slip boundary conditions were used. x, y, and z represent the components in the three directions of the rectangular coordinate system. The blood flow satisfies the Navier-Stokes (N-S) equation and the continuity equation:

$$\frac{D\nu_{x}}{Dt}=-\frac{1}{\rho }\frac{\partial P}{\partial x}+\frac{\mu }{\rho }\left(\frac{\partial^{2}\nu_{x}}{\partial x^{2}}+\frac{\partial^{2}\nu_{x}}{\partial y^{2}}+\frac{\partial^{2}\nu_{x}}{\partial z^{2}}\right)$$ (1)

$$\frac{D\nu_{y}}{Dt}=-\frac{1}{\rho }\frac{\partial P}{\partial y}+\frac{\mu }{\rho }\left(\frac{\partial^{2}\nu_{y}}{\partial x^{2}}+\frac{\partial^{2}\nu_{y}}{\partial y^{2}}+\frac{\partial^{2}\nu_{y}}{\partial z^{2}}\right)$$ (2)

$$\frac{D\nu_{z}}{Dt}=-\frac{1}{\rho }\frac{\partial P}{\partial z}+\frac{\mu }{\rho }\left(\frac{\partial^{2}\nu_{z}}{\partial x^{2}}+\frac{\partial^{2}\nu_{z}}{\partial y^{2}}+\frac{\partial^{2}\nu_{z}}{\partial z^{2}}\right)$$ (3)

$$\frac{\partial \nu_{x}}{\partial x}+\frac{\partial \nu_{y}}{\partial y}+\frac{\partial \nu_{z}}{\partial z}=0$$ (4)

In the equations, ρ represents the density of blood; $\nu_{x}{,\nu }_{y}{,\nu }_{z}$ are the components of blood flow velocity; μ is the dynamic viscosity; and P is pressure. The density is 1,060 kg/m^3^, and the dynamic viscosity is 0.0035pas.

The flow velocity of the SVC and IVC were extracted from the previous TCPC patient’s echocardiography and recorded during a respiratory cycle, and the measured images of the SVC and IVC flows were extracted into a flow curve that changes with the respiratory cycle. The computing cycle was 2.54s, the time step was 0.001 s. The results obtained by the average of 2,540 time steps.

The flow velocity curve was applied to the inlets of the SVC and IVC. The conditions at light, moderate, and heavy exercise were simulated by setting the IVC flow velocity at 2, 3, and 4 times the IVC flow velocity at rest, respectively, and reducing the resistance by 5%, 10%, and 15%, respectively. In addition, the SVC flow velocity for heavy exercise was set 50% higher than the SVC flow velocity at rest, and the SVC flow velocity for light and moderate exercise were set the same as the SVC flow velocity at rest (Marsden et al., 2009).

### 2.4 Computation of energy efficiency

Based on the energy efficiency formula, the energy efficiency of the flow from inlet to outlet was computed for different levels of exercise. Because of the same SVC and IVC configurations at all inlets and the identical pulmonary artery configurations at all outlets, the difference in energy efficiency was mainly due to the different configurations of the extracardiac conduits. The energy efficiency satisfies the following equation:

$$\begin{array}{c} E_{diss}=-\overset{N_{in}}{\underset{i=1}{\sum }}{ʃ}_{Ai}\left(p+\frac{1}{2}\rho u^{2}\right)u∙dA\\ -\overset{N_{out}}{\underset{i=1}{\sum }}{ʃ}_{Ai}\left(p+\frac{1}{2}\rho u^{2}\right)u∙dA, \end{array}$$ (5)

where u is the flow velocity, p is the pressure, ρ is the density, $N_{in}$ and $N_{out}$ are the number of inlets and outlets of the model, respectively, and $A_{i}$ is the area of the i-th inlet or outlet. Energy efficiency is the ratio of the second term to the first term in the above equation.

### 2.5 Particle tracking

This method was used to count the left pulmonary artery (LPA) and right pulmonary artery (RPA) flow of the IVC during a whole respiratory cycle. Assuming that the particles have no mass, and the diffusion effect is ignored, in a single breathing cycle, about 400 particles are released every 0.001 s. The total amount of released particles in the whole cycle is 1016000, and the total amount of captured particles is 936000. The uncapped particles are particles still flowing in the fluid domain at the end of breathing, and the captured particle mass at the LPA and RPA outlets can be counted to obtain the left and right blood diversion ratio.

### 2.6 Postoperative data collection

The patient had been followed up for 3 years, the radionuclide lung perfusion, CTA, cardiovascular angiography and catheterization were performed 12 months after operation, echocardiography was performed every year.

Radionuclide lung perfusion with technetium-99 m (99 mTc, Atomic High Technology Co., Ltd., Beijing, China)-labeled macroaggregated albumin (MAA, Jiangsu Institute of Nuclear Medicine) was performed to determine the distribution of the patient’s IVC flow. The tracer, 99 mTc-labeled MAA, was administered through the dorsal vein of the foot. The distribution of IVC flow to the left and right lungs was compared.

#### 2.6.1 Imaging method

After a slow intravenous injection of 99 mTc-labeled MAA in the supine position, the patient was scanned at eight views, including anteroposterior, left anterior oblique, left lateral, left posterior oblique, posteroanterior, right posterior oblique, right lateral and right anterior oblique views.

#### 2.6.2 Quantitative analysis

The posteroanterior image was used to measure the radionuclide counts in the left and right lungs.

## 3 Results

We obtained the preoperative CTA data of the patient and then created a model. Different spatial structures were used to connect the extracardiac conduit and pulmonary artery. These spatial structures were divided into 4 groups. The experimental results are described as follows:

### 3.1 Pressure gradient

The PG was calculated by subtracting the sum of pressures at the left and right pulmonary hilum by the sum of pressure of SVC and IVC, and then divided by 2.

**FIGURE 1:**
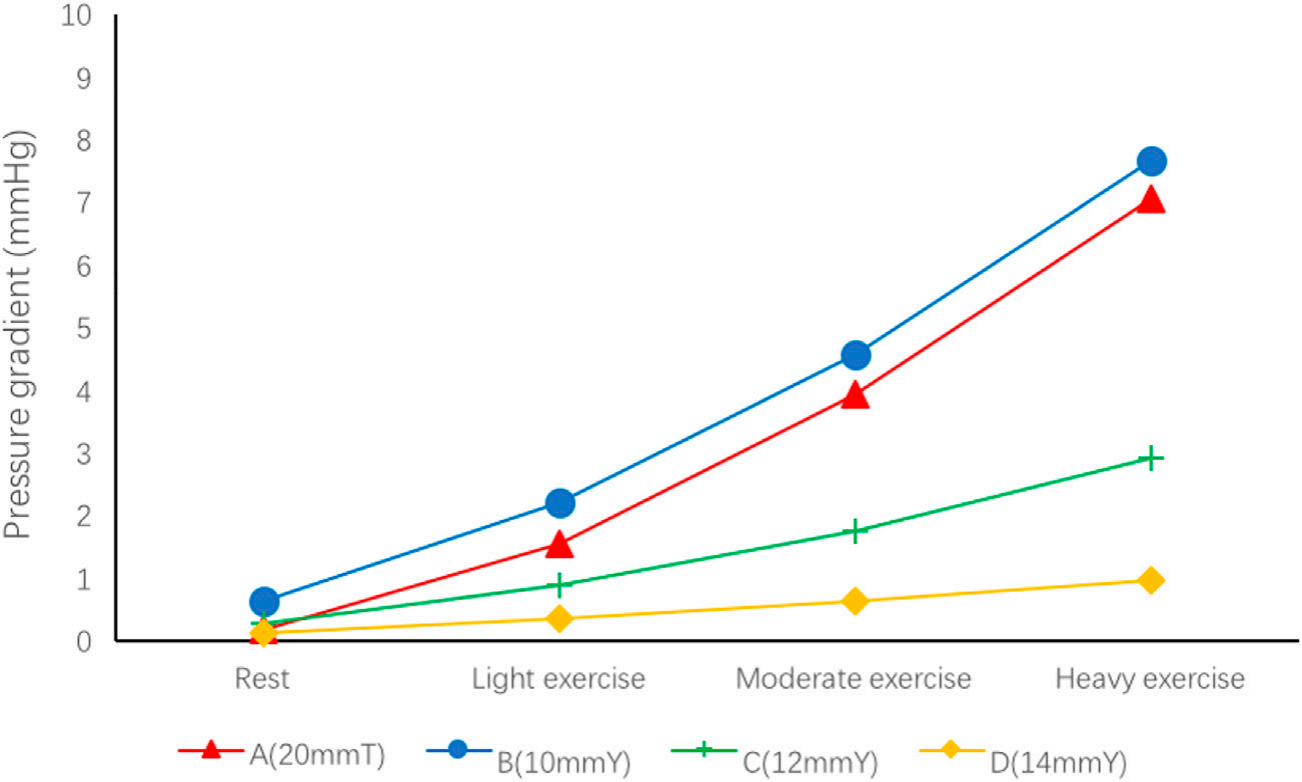
Pressure gradient in the four groups of different spatial configurations.

The PG of each group is lower than 1 mmHg at rest, but with the increase of exercise, the PG gradually increases, and in group A, PG can reach 7.07 mmHg under heavy exercise. For Y-shaped conduit, the highest PG is 7.67 mmHg in group B (10 mmY). The increase of branch diameter can effectively reduce the PG under different levels of exercise. The PG in Group D was lowest and the increase rate was the slowest under rest and different levels of exercise (Figure 1). Therefore, if the branch diameter of Y-shaped conduit is not enough, the PG would be higher.

**FIGURE 2:**
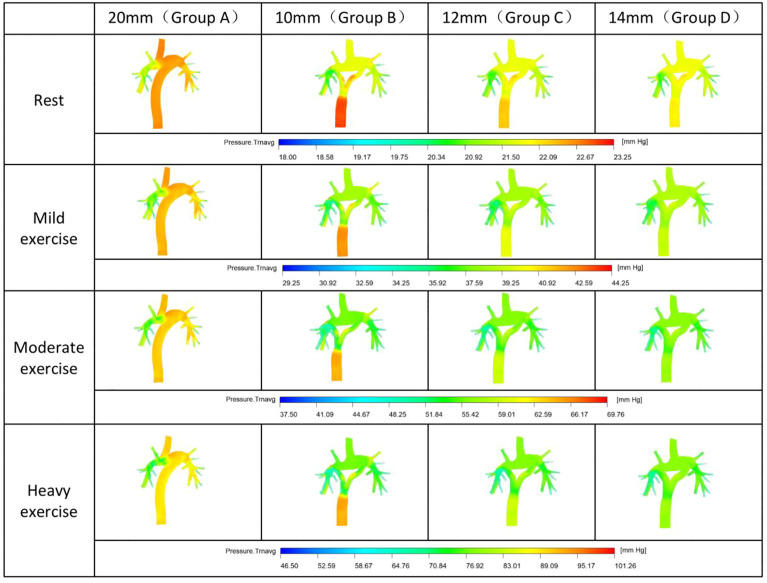
Nephogram of pressure distribution.

We look for the main source of the PG by the pressure distribution nephogram (Figure 2). In group A, the main source of PG was located between the intersection of SVC and the right hilum. For Y-shaped conduit, PG mainly comes from the bifurcation of conduit. However, the PG gradually decreases with the increase of bifurcation diameter, and the PG almost cannot be found in group D (14 mmY).

### 3.2 Flow velocity

The flow velocity was calculated at bifurcation of conduit in group B, C and D, and at the same section of T-shaped conduit trunk in group A.

**FIGURE 3:**
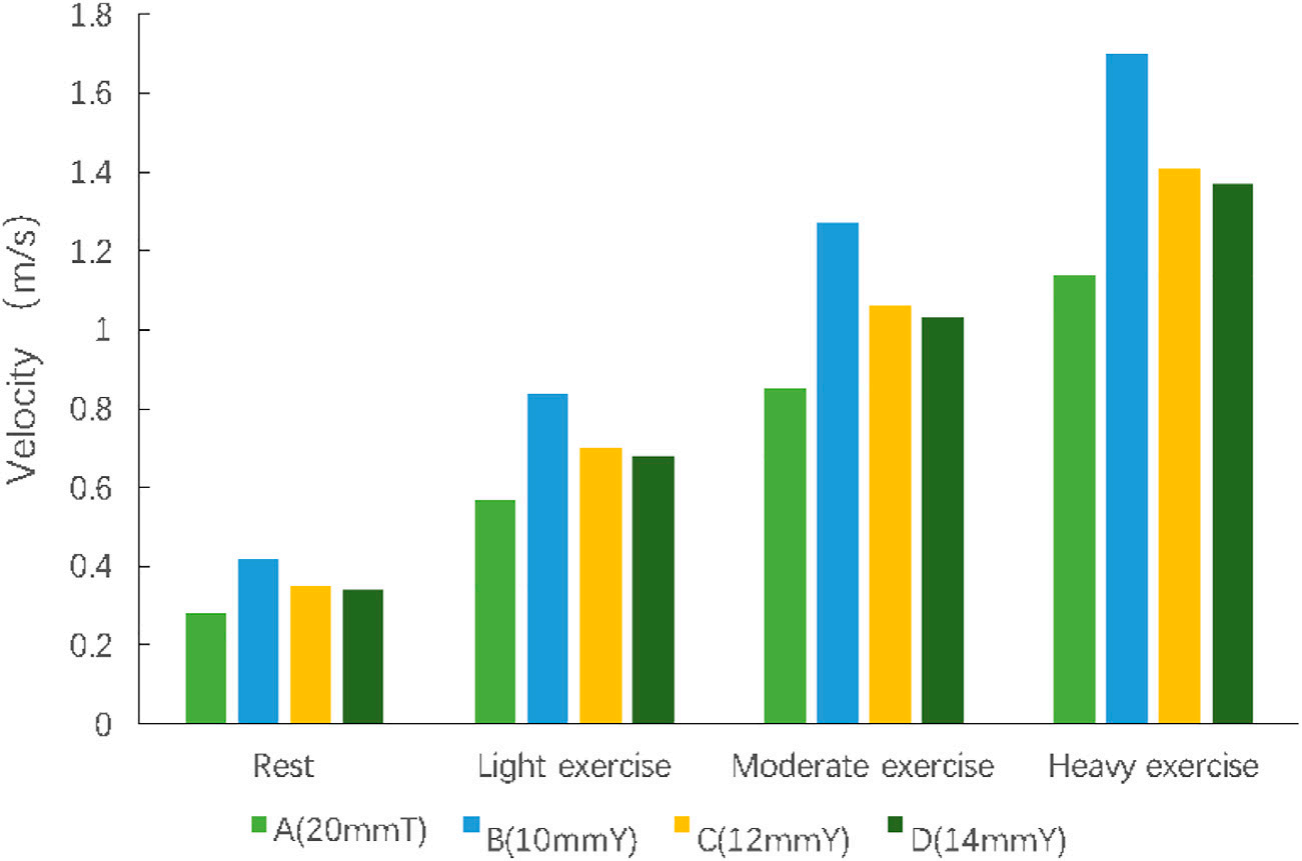
The velocity at corresponding section of each group.

At the inlet of the SVC and IVC in each group were same. At rest and different levels of exercise, group B had a significantly faster flow velocity in the Y-graft branches than other groups. As the branch diameter increased, the branch flow velocity gradually decreased (Group C). The branch flow velocity in Group D was basically same as that of the trunk of the conduit (Figure 3).

Therefore, a smaller branch diameter might be the main reason of the faster flow for Y-shaped conduit.

### 3.3 Distribution of IVC flow

The distribution of the IVC flow to the LPA and RPA is a main determinant for prognosis of Fontan circulation. We were able to determine those different spatial configurations which significantly changed the distribution of the IVC flow to the LPA and RPA by the particle tracking. We assumed that the ratio of LPA and RPA distribution was 45 to 55 (LPA/RPA) (Marsden et al., 2009).

**FIGURE 4:**
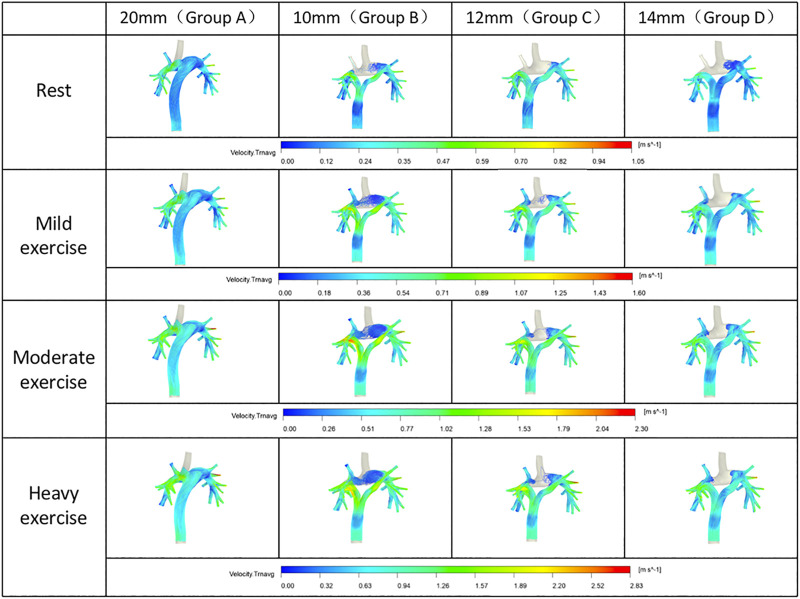
Distribution of the inferior vena cava blood flow.

The conventional surgical method (Group A) alleviated the competition between the SVC and IVC flows, but the misalignment of the anastomosis caused more blood in the IVC flow to the LPA (60%) (Figure 4). However, the distribution percentage of IVC blood in LPA and RPA changed dramatically (LPA, 46.4%-60%) from rest to exercise. The IVC flow distribution was more balanced for the group B, while that in group D was the most stable during different levels of exercise (Table 1).

**TABLE 1:** Distribution of the inferior vena cava blood flow under different levels of exercise.

Percentage (%)	A (20 mm T)		B (10 mm Y)		C (12 mm Y)		D (14 mm Y)	
	L	R	L	R	L	R	L	R
Rest	60	40	43.4	56.6	42	58	40	60
Light exercise	48.2	51.8	42.4	57.6	42.3	57.7	40.3	59.7
Moderate exercise	46.4	53.6	42.8	57.2	41.1	58.9	40.8	59.2
Heavy exercise	47	53	40.7	59.3	40.7	59.3	40.6	59.4

### 3.4 Energy efficiency

**FIGURE 5:**
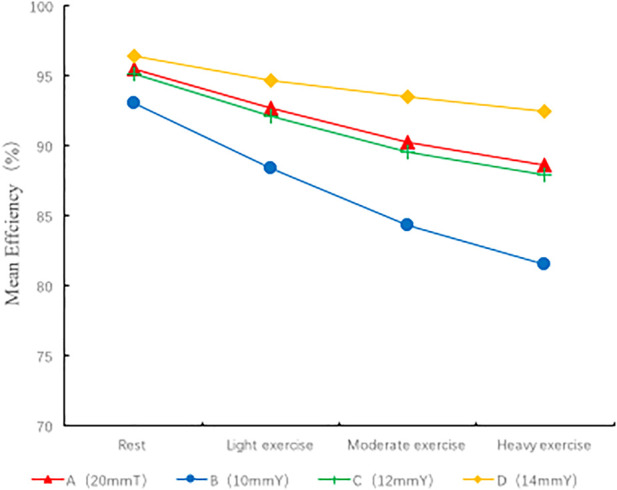
Energy efficiency of different spatial configurations.

The energy efficiency of each geometric configuration was computed according to the energy efficiency equation (Figure 5). In all models, group B (10 mmY type) was associated with the lowest energy efficiency (93% at rest, 81.5% for heavy exercise), and the energy efficiency was 11.5% lower during heavy exercise than at rest. Compared with the conventional group A (20 mmT), the Y-graft branches (10 mmY) of group B did not significantly improve the energy efficiency of the surgical method. However, the diameter of the Y-graft branches directly affected the overall energy efficiency. As the branch diameter increased, the energy efficiency gradually improved. Group D (14 mmY) was associated with the highest energy efficiency (96.4% at rest, 92.4% for heavy exercise), and the energy efficiency was only 4% during heavy exercise than at rest. The energy efficiency in all groups was significantly lower during exercise than at rest. Therefore, a large diameter of the Y-graft branches can significantly improve the energy efficiency of YCPC, while a smaller branch diameter increases the energy consumption (Figure 5).

### 3.5 Application of a handcrafted Y-shaped conduit

To facilitate AI learning in the future, we try to quantify the simulation indicators to select the optimal procedure. According to the best to the worst indicators, we score each indicator of 4, 3, 2 and 1 respectively (Table 2).

**TABLE 2:** The scores of each indicator for 4 groups.

Score	PG	Velocity	Energy efficiency	Balanced IVC flow	Total
A (20 mm T)	2	4	2	1	9
B (10 mm Y)	1	1	1	4	7
C (12 mm Y)	3	2	3	3	11
D (14 mm Y)	4	3	4	2	13

PG, Pressure gradient; IVC, inferior vena cava.

In the operation design stage, based on Table 2, the group D (14 mm Y) had following advantages: the lowest PG, the lower velocity at bifurcation of Y-shaped conduit, the highest energy efficiency, and a relatively balanced (not the most balanced, but the most stable) IVC flow distribution.

**FIGURE 6:**
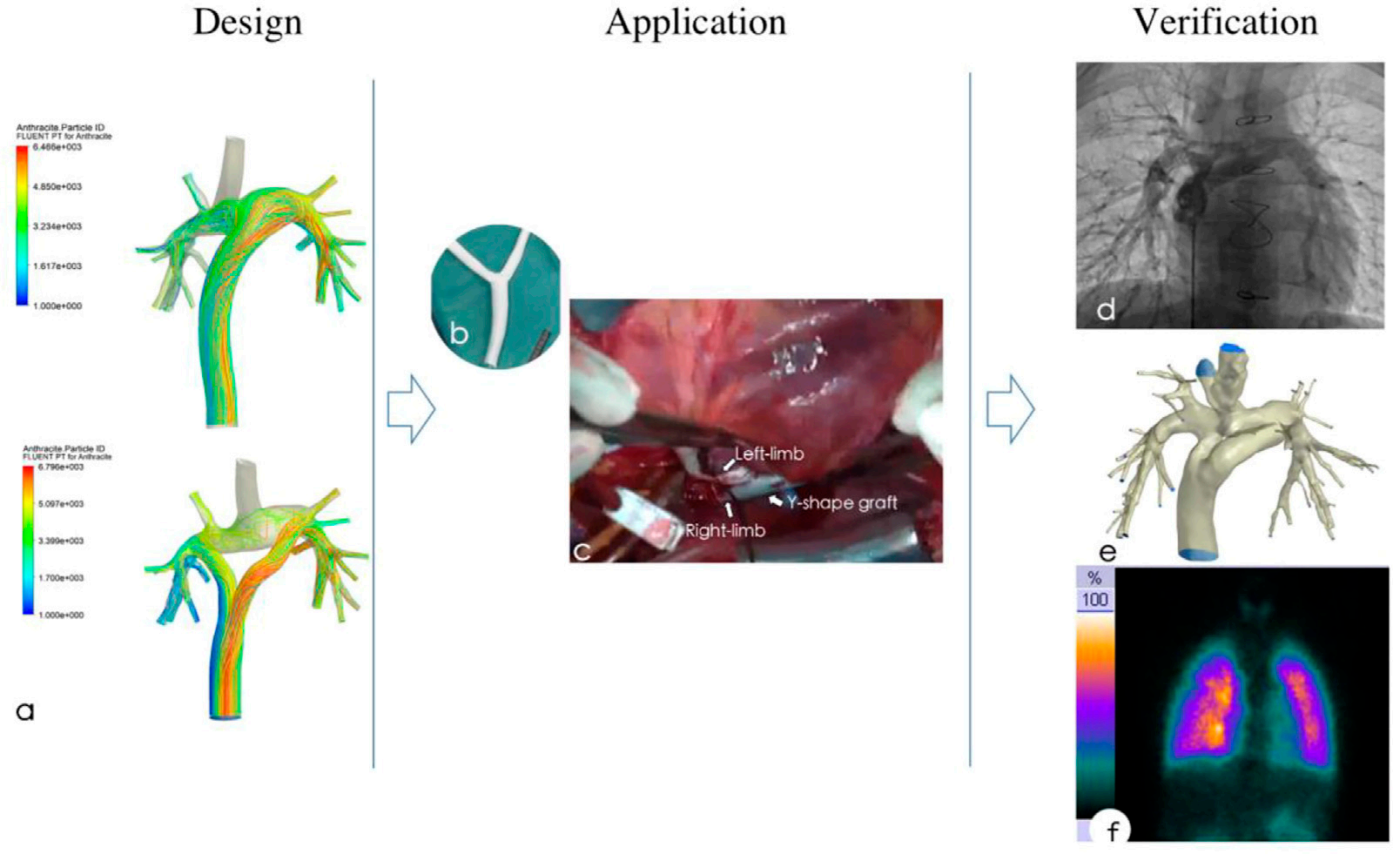
Preoperative design based on computational fluid dynamics simulation **(A)**, then a handcrafted Y-shaped conduit **(B)** was applicated for functional single ventricle **(C)**, postoperative catheter angiography **(D)**, CT angiography **(E)** and pulmonary perfusion scan **(F)** showed normal pulmonary pressure and resistance, while IVC flow was distributed uniformly.

In the application stage, the right branch of the Y-shaped conduit was moved to a position close to the midline because the right superior pulmonary artery could not obtain IVC blood flow. Subsequently, a handcrafted Y-shaped conduit (20 mm-14mm-14 mm) was used for the YCPC procedure under mild hypothermic cardiopulmonary bypass, because the commercially available Y-grafts was diameter-preserving (20 mm-10 mm-10 mm). The procedure went well and postoperative recovery was good (Figure 6).

### 3.6 Verification

In the postoperative stage, tracheal extubation was conducted on the 7th day after surgery. The patient was discharged from hospital on the 15th day after surgery. The patient had been followed up for 3 years with a good recovery. During the follow-up, the echocardiography, cardiovascular CTA, pulmonary blood perfusion scan, cardiovascular angiography and catheterization were perfomed to verification the outcome (Figure 6).

#### 3.6.1 Morphology and velocity

The CTA showed that the conduit was in good shape, and there was no stenosis at each anastomosis; And cardiovascular angiography showed that the Y-graft extracardiac conduit made the IVC blood evenly distribute to the LPA and RPA, which brought good blood perfusion in pulmonary (Supplementary Video S1).

The echocardiography was performed every year regularly after the operation, and the velocity of each anastomosis were specifically detected. We recorded them during the follow-up period. Echocardiography showed that the flow velocity at the anastomotic site between the IVC and RPA was 0.35 m/s; The velocity of the anastomosis between the conduit and IVC, LPA and RPA was 0.3 m/s, 0.48 m/s and 0.45 m/s respectively, which was basically consistent with the velocity before operation (0.24–0.41 m/s).

#### 3.6.2 PG

Catheterization showed there is no PG in the conduit and pulmonary, the pressure of vena cava and pulmonary both are 24/16 mmHg, while the pulmonary vascular resistance was 1.18 woods.

#### 3.6.3 Distribution of the IVC

We used the distribution ratio of IVC flow in both lungs to represent the distribution of hepatic blood flow, and achieved good results. Pulmonary blood perfusion scan was used to detect the distribution of the IVC flow to LPA and RPA. The 99 mTc was injected through the dorsal vein of the foot to show the distribution of the IVC flow to LAP and RPA. The results showed that the IVC flow was distributed uniformly to LPA and RPA (46.71 vs. 53.29) and was uniformly distributed to the upper, middle and lower parts of the lung. The result was similar as the preoperative simulation data (Table 3). In addition, the hepatic function (transaminase and bilirubin) was normal at the early stage and during the follow-up period.

**TABLE 3:** Percentage of inferior vena cava flow by radionuclide lung perfusion.

Percentage (%)	Left	Right
Upper	8.1	9.66
Middle	21.79	25.97
Lower	16.82	17.66
Total	46.71	53.29

## 4 Discussion

After nearly 50 years of development and improvement of the series of Fontan procedure, its clinical efficacy has continuously improved. In 1988, de Leval de Leval et al. (1988) found that conventional Fontan procedures could easily cause intra-atrial vortexes to increase energy consumption and thus lead to resistance to forward blood flow. Computer simulations has proven the importance of linear blood flow in blood vessels. Thus, TCPC was designed to provide linear flow in blood vessels to improve the conventional Fontan procedures.

With the continuous innovation of technology, data modeling and CFD research based on CT and MR imaging revealed that TCPC is still problematic in high energy consumption and uneven IVC flow split (Sundareswaran et al., 2012; Tang et al., 2013). Some researchers have applied CT-based vascular geometric models in individual patients. From the perspective of fluid mechanics, they proved that the use of a Y-graft extracardiac tube to connect the SVC to the pulmonary arteries and another Y-graft extracardiac tube to connect the pulmonary arteries to the IVC can form a diamond-shaped spatial configuration to optimize the conventional TCPC (Soerensen et al., 2007). Although this design is theoretically feasible and can make blood flow distribution more evenly, it cannot be effectively implemented from a surgical perspective. Moreover, we believe that if the competition between the SVC and IVC flows cannot be avoided, energy consumption can easily be high. Subsequent studies show that split of the blood flow in the IVC into two flows can make the blood evenly distributed and thus increase fluid efficiency and reduce vortex and conduit wall shear force (Kanter et al., 2012; Martin et al., 2015). In theory, YCPC brings two major advantages, decreases in energy consumption and even IVC flow split. Therefore, YCPC may gradually replace conventional TCPC in clinical applications.

The clinical effect of Fontan series surgery is improving, but the two desired theoretical advantages of YCPC design have not been fully realized in clinical application in view of the current studies. There might be higher resistance and less balanced hepatic flow for YCPC than lateral tunnel or extracardiac conduit (Martin et al., 2015; Trusty et al., 2016).

Our center had conducted a long-term and in-depth clinical study on a series of Fontan procedures since 1980. We reported the first group of traditional modified Fontan operation and TCPC of China in 1984 and 1992, respectively (Wang et al., 1984; Wang et al., 1992). The subsequent follow-up studies on pulmonary vascular perfusion and activity tolerance were carried out (Yin et al., 2006; Yin et al., 2009; Yin et al., 2012)^,^ and the longest follow-up period was 32 years (Zhang et al., 2020). In 2015, we reported the first group of YCPC of China with good follow-up result of hemodynamics (Wang et al., 2015). We found that the individualized differences of patients significantly affected the postoperative efficacy. Therefore, a good clinical operation design and effect prediction is urgently needed.

In previous studies (Soerensen et al., 2007; Sundareswaran et al., 2009), CFD technology were applied for simulation of the hemodynamics of TCPC, but they were not used for the optimization of spatial structures and prediction of efficacy. Moreover, there were huge defects in their applications, such as incomplete image information of pulmonary arteries. Although many studies have been done, it is impossible to simulate blood flow realistically, and it is impossible to predict the impact of the main branches of the pulmonary artery on blood flow energy consumption, resulting in a significantly poorer clinical prognosis than expected.

Because commercial Y-grafts currently used are diameter-preserving, in order to make the extracardiac conduits more efficient, some international centers (Marsden et al., 2009; Yang et al., 2015) began to use hand-sewn Y-grafts (extracardiac conduits) to ensure the sum of the area of the two branches close to the area of the trunk. In their studies, they found that the efficiency of the diameter-preserving Y-grafts was 88.5%, while the efficiency of area-preserving Y-grafts could reach 90.3%. In the case of exercise, the difference between the efficiencies of diameter-preserving Y-grafts and area-preserving Y-grafts is more obvious. Therefore, we used different Y-graft designs in our study to analyze their differences.

Our study has shown that when compared with TCPC, the YCPC can achieve more evenly distribution of the SVC and IVC flows to the left and right lungs, resulting in a balanced distribution of the IVC flow and promotes the haptic factors to flow into the left and right lungs, and reducing the incidence of postoperative pulmonary arteriovenous fistula (de Zelicourt et al., 2011). In the application of the Y-grafts, the branch diameter directly affects the branch flow velocity. The diameters of Y-grafts in the diameter-preserving group were 20-10-10 mm. The branch flow velocity increased significantly in the beginning. This in turn leads to an increase in energy consumption. As the branch diameter increases, the rate in the beginning of the branch also decreases, thus reducing the energy consumption of the conduits. In the area-preserving group (20-14-14 mm), the sum of the cross-sectional area of the two branches were approximately equal to the cross-sectional area of the trunk, the flow velocity in the beginning of the branch was gradually reduced to close to that of the trunk. The branch diameter of the Y-graft directly affected the pressure at the inlet of the IVC, and compared with the conventional TCPC group, the pressure at the IVC inlet in the diameter-preserving group (20-10-10 mm) was significantly higher. With increase of the branch diameter, the pressure at the IVC inlet gradually decreased. The pressure at the IVC inlet in the area-preserving group (20-14-14 mm) was close to that in the conventional TCPC group. Overall, compared with the conventional spatial configuration of TCPC, the area-preserving spatial configuration of the YCPC were not significantly different in the pressure at the SVC and IVC inlets and the overall flow velocity but resulted a more even distribution of the IVC flow to the left and right lungs without increasing energy consumption.

We found that smaller branch diameter may be the main reason for the PG in Y-shaped conduit which will bring more energy consumption. Sufficient cross-sectional area of branch brought a higher energy efficiency. This theory might explain why the resistance for commercially available YCPC was higher (Trusty et al., 2016).

### 4.1 Study limitations

This study is only a preliminary study, providing a possibility for future AI learning. We need more cases and studies to provide objective data for machine learning.

In addition, it has great limitations when addressing clinical application. There are many factors can bring decreased energy efficiency such as wall shear stress, vortex, etc. The mechanism of higher energy efficiency did not be study and discuss in this research. Furthermore, Blood vessels were assumed to be rigid structures, but they are elastic structures that can be deformed. FSI (Fluid Structure Interaction) can simulate the interaction between fluid and solid, so as to simulate dynamic blood vessels, which is closer to the real clinical application. These will be discussed in future studies.

## 5 Conclusion

By combining the advantages of clinical medicine, radiodiagnostics, and fluid mechanics, we analyzed the optimal spatial configuration of the extracardiac conduit suitable for TCPC. We found that the smaller bifurcation diameter may be the main reason for the higher PG and faster velocity in Y-shaped conduit. A good preoperative design on individual basis based on CFD could bright more balance IVC distribution and higher energy efficiency. This study also provides a theoretical basis for clinical applications of YCPC.

## Data Availability

The original contributions presented in the study are included in the article/Supplementary Material, further inquiries can be directed to the corresponding author.

## Supplementary material

SUPPLEMENTARY VIDEO S1 Preoperative design based on computational fluid dynamics simulation and postoperative catheter cardiovascular angiography of Y-shaped conduit total cavopulmonary connection.

## Abbreviations

CFD, Computational fluid dynamics; CTA, Computed tomography angiography; FSV, Functional single ventricle; IVC, Inferior vena cava; LPA, Left pulmonary artery; PG, Pressure gradient; RPA, Right pulmonary artery; TCPC, Total cavopulmonary connection; YCPC, Y-shaped conduit total cavopulmonary connection
